# Nonresolving Neuroinflammation Regulates Axon Regeneration in Chronic Spinal Cord Injury

**DOI:** 10.1523/JNEUROSCI.1017-24.2024

**Published:** 2024-11-07

**Authors:** Andrew N. Stewart, Christopher C. Bosse-Joseph, Reena Kumari, William M. Bailey, Kennedy A. Park, Victoria K. Slone, John C. Gensel

**Affiliations:** ^1^Department of Neuroscience, University of Kentucky, Lexington, Kentucky 40536; ^2^Spinal Cord and Brain Injury Research Center, University of Kentucky, Lexington, Kentucky 40536; ^3^College of Medicine, University of Kentucky, Lexington, Kentucky 40536; ^4^Department of Physiology, University of Kentucky, Lexington, Kentucky 40536

**Keywords:** axon regeneration, chronic spinal cord injury, gene therapy, inflammation, macrophage depletion, PLX-5622, PTEN, retrograde AAV

## Abstract

Chronic spinal cord injury (SCI) lesions retain increased densities of microglia and macrophages. In acute SCI, macrophages induce growth cone collapse and facilitate axon retraction away from lesion boundaries. Little is known about the role of sustained inflammation in chronic SCI or whether chronic inflammation affects regeneration. We used the colony-stimulating factor-1 receptor inhibitor, PLX-5622, to deplete microglia and macrophages months after complete crush SCI in female mice. Transcriptional analyses revealed a significant inflammatory depletion within chronic SCI lesions after PLX-5622 treatment. Both transcriptional analyses and immunohistochemistry revealed that Iba1^+^ cells repopulate to predepleted densities after treatment removal. Neuronal-enriched transcripts were significantly elevated in mice after inflammatory repopulation, but no significant effects were observed with inflammatory depletion alone. Axon densities also significantly increased within the lesion after PLX-5622 treatment and after repopulation. To better examine the effect of chronic inflammation on axon regeneration, we tested PLX-5622 treatment in neuronal-specific phosphatase and tensin homolog protein (PTEN) knock-out (PTEN-KO) mice. PTEN-KO was delivered using spinal injections of retrogradely transported adeno-associated viruses (AAVrg's). PTEN-KO did not further increase axon densities within the lesion beyond the effects induced by PLX-5622. Axons that grew within the lesion were histologically identified as 5-HT^+^ and CGRP^+^, both of which are not robustly transduced by AAVrg's. Our work identified that increased macrophage/microglial densities in the chronic SCI environment may be actively retained by homeostatic mechanisms likely affiliated with a sustained elevated expression of CSF1 and other chemokines. Finally, we identify a novel role of sustained inflammation as a prospective barrier to axon regeneration in chronic SCI.

## Significance Statement

Inflammatory macrophages infiltrate the spinal cord after a spinal cord injury (SCI) and remain at elevated densities around the lesion chronically. Little is known about the consequences of sustained spinal inflammation in chronic SCI, but it is known that macrophages impair regeneration early after SCI. We hypothesized that persistent neuroinflammation may function as a sustained barrier that chronically impairs axon regeneration. Our work identifies that the chronic SCI environment actively maintains increased densities of macrophages and implicates sustained inflammation as a persistent barrier to repair. Findings from our work change our understanding of the maintenance of nonresolving inflammation in chronic SCI and identify a need to focus on overcoming chronic inflammatory barriers that limit repair after neurotrauma.

## Highlights

- Macrophages and microglia repopulate the chronically injured spinal cord after depletion.
- Colony-stimulating factor-1 receptor (CSF1R) antagonism in chronic spinal cord injury augments the growth of specific axon types in the lesion.
- CSF1R antagonism does not augment a phosphatase and tensin homolog protein knock-out–induced functional recovery.

## Introduction

Macrophage recruitment into the lesion core is a major regulator of spinal cord injury (SCI) and recovery responses (Kigerl et al., 2009; Gensel et al., 2012; Zhang et al., 2015, 2016). In acute SCI, macrophages exacerbate axon damage through direct physical interactions and also facilitate axon die-back away from the lesion boundaries through the secretion of matrix metalloproteases (MMPs) that induce growth cone collapse and axon retraction (Horn et al., 2008; Busch et al., 2009). Indirectly, macrophages restrict axon growth and regeneration by orchestrating the formation of the glial scar (Bellver-Landete et al., 2019; Zhou et al., 2020; Kisucká et al., 2021). Collective evidence points to inflammation as a major barrier to regeneration through the lesion via both direct and indirect mechanisms.

Signaling from macrophages orchestrates many of the glial scarring components, including the recruitment of fibroblasts into the lesion (Zhu et al., 2015) and the activation of both astrocytes and microglia (Zhou et al., 2020; Kisucká et al., 2021). Each of these cells persists at increased densities around chronic lesions and contributes to the production of a growth inhibitory extracellular matrix (Inman and Steward, 2003; Jones et al., 2003; Filous et al., 2014). Chronic stages after SCI are hallmarked by stabilization of functional recovery and with very little continued resolution of the lesion environment (Beck et al., 2010; Kwiecien et al., 2020).

Interventions aiming to induce axon growth and regeneration after SCI exhibit significantly less efficacy when interventions are delayed even 1 week postinjury which corresponds to a timepoint of peak macrophage infiltration into the spinal cord (Beck et al., 2010; Blesch et al., 2012). Very little is known about the role of nonresolving neuroinflammation around chronic SCI lesions or the effects of chronic neuroinflammation on axon regeneration with, or without, growth-promoting interventions. The proceeding experiments sought to determine if persistent inflammation around and within SCI lesions functions as an inhibitory barrier to axon growth in chronic SCI.

We identified nonresolving inflammation as a hallmark of chronic lesions which is susceptible to macrophage/microglial depletion by treatment with the colony-stimulating factor-1 receptor (CSF1R) antagonist, PLX-5622 (PLX). We combined PLX with concurrent regenerative stimulation using retrogradely transported adeno-associated virus (AAVrg's) to knock out the phosphatase and tensin homolog protein (PTEN) to facilitate axon growth. We observed a significant increase in axon densities within the lesion after chronic PLX treatment. However, the phenotype of axons that grew into the lesion were not those affected by AAVrg's or PTEN-KO. Axons that expanded in the lesion were observed to be in part 5-HT^+^, but largely CGRP^+^ fibers. Collectively, we present novel data that support inflammation within the chronically injured spinal cord as a regulator of regeneration of specific axon fiber types within the lesion.

## Materials and Methods

### Experimental design

#### Defining the chronic SCI timeline and lesion environment

Transcriptional analysis using the NanoString Neuropathology panel (XT-CSO-MNROP1-12; NanoString Technologies) was performed to determine a plateau in inflammatory resolution within the chronically injured spinal cord. We evaluated mRNA from spinal cord homogenates in either naive (*n* = 6) mice or at 1 week (*n* = 6), 2 weeks (*n* = 5), 9 weeks (*n* = 3), and 12 weeks (*n* = 3) postinjury. Prior reports have indicated that a resolution of inflammation reaches a plateau between 9 and 12 weeks post-SCI in rodents (Beck et al., 2010; Kwiecien et al., 2020) which was the basis of our chosen timepoints.

#### Determining the ability of PLX to deplete macrophages/microglia in chronic SCI

Mice were treated with PLX-5622 (PLX; C-1521; Chemgood; 1,200 ppm) via dietary feed (prepared at Envigo), for either 9 (*n* = 5), 14 (*n* = 4), or 21 d (*n* = 4) post-SCI. A PLX dose of 1,200 ppm was chosen based on prior literature (Henry et al., 2020). Furthermore, we included both a normal diet as a vehicle control (*n* = 5), and a repopulation group that was treated for 21 d before allowing 3 weeks of macrophage/microglial repopulation by removing PLX (*n* = 4). For this first experiment, mice were treated with PLX beginning at 11 weeks post-SCI, except for one vehicle, and one mouse treated for 9 d which started treatment at 7 weeks post-SCI and were used as pilot to explore treatment efficacy. These mice were combined in the groups above due to the successful depletion caused by PLX.

#### Determine the effects of chronic PLX administration on the lesion environment

Mice were treated with either vehicle (*n* = 6), 14 d of PLX (*n* = 6), or 14 d of PLX with 21 d of repopulation (*n* = 4). For this experiment, mice began treatment at 7 weeks post-SCI and were killed at either 9 weeks or 12 weeks postinjury. For vehicle-treated mice, *n* = 3 were killed at 9 weeks, and the other *n* = 3 were killed at 12 weeks post-SCI to control for potential differences in the chronic injury environment. Importantly, we did not detect significant differences between 9 and 12 weeks post-SCI in any mRNA transcripts quantified after false discovery rate (FDR) correction; therefore, vehicle-treated mice from both time points were combined for analysis as a single chronic SCI group.

#### Combining PTEN-KO with PLX in chronic SCI to determine the role of macrophages/microglia on axon regeneration

**Figure 1.**
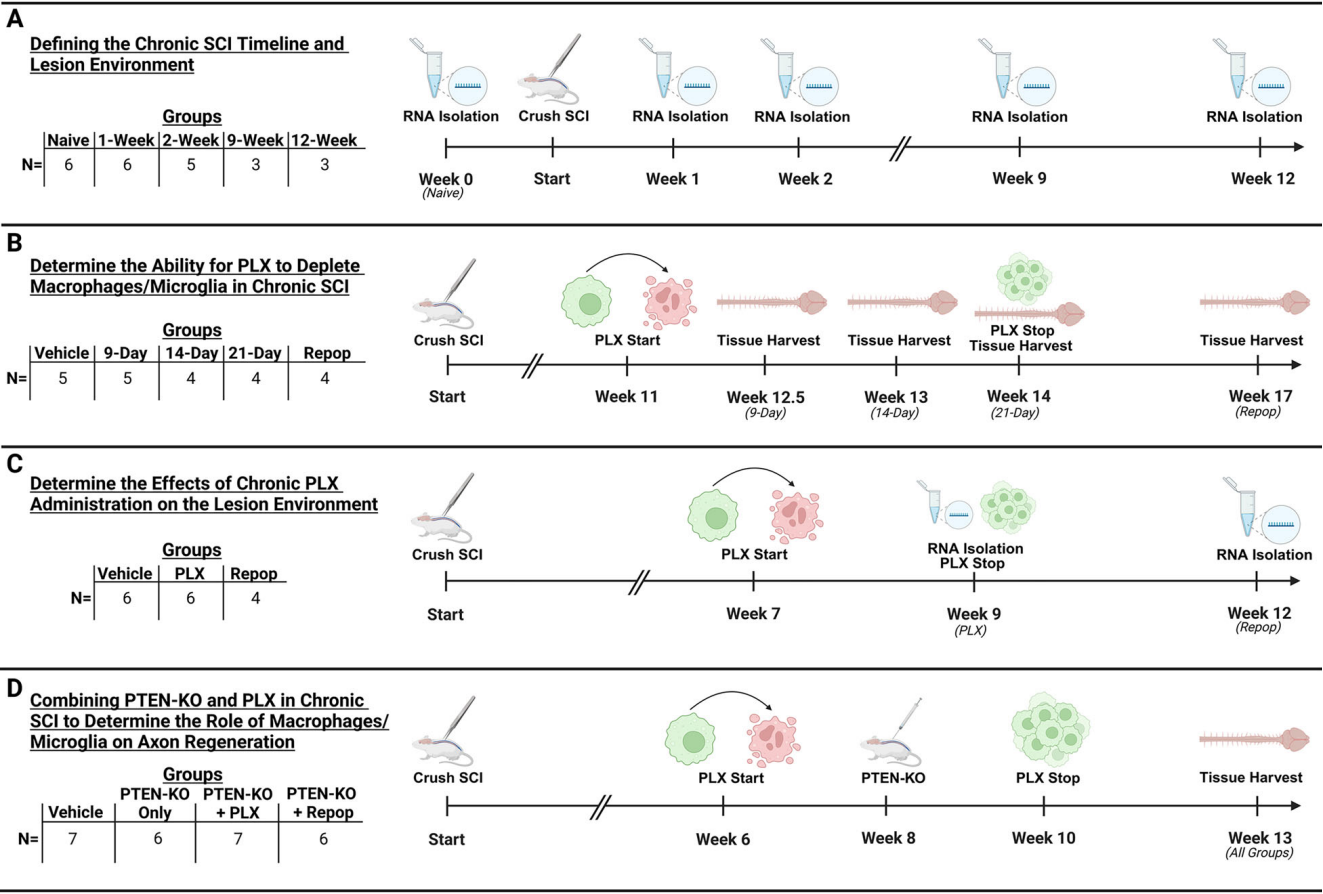
Groups, sample sizes, and timeline of experiments. In total, four experiments were performed to obtain either RNA for transcriptional analysis (***A***, ***C***) or tissue for immunohistochemistry (***B***, ***D***). In our first experiment (***A***), tissue was obtained from half the naive mice at the time of tissue harvest for our 9- and 12-week post-SCI timepoints to serve as age-matched controls. In our second experiment (***B***), tissue was obtained from one vehicle-treated mouse at each tissue harvest time point, with tissue obtained from one other mouse at 9 weeks post-SCI during a pilot experiment. In our third experiment (***C***), RNA was obtained from three vehicle-treated mice at Week 9 and the remaining three at Week 12 to control for time post-SCI. In our final experiment (***D***), tissue was harvested from all mice at 13 weeks post-SCI. This figure was created with BioRender.com.

To get a better understanding of the role of chronic inflammation on axon regeneration, we advanced the following groups to combine inflammatory depletion/repopulation with regenerative stimulation: vehicle (group name, vehicle; no PTEN-KO and no PLX; *n* = 7), PTEN-KO/only (group name, PTEN-KO; *n* = 6), PTEN-KO/PLX (group name, PLX; sustained for 7 weeks during the experiment; *n* = 6), and PTEN-KO/repopulation (group name, Repop; received 28 d of PLX prior to PTEN-KO and then removed from PLX treatment for 21 remaining days of the study; *n* = 7). Mice were given PLX beginning 6 weeks post-SCI, AAVrg's to deliver PTEN-KO, or RFP alone at 8 weeks post-SCI and removed from PLX at 10 weeks post-SCI for the repopulation group. Mice were allowed 5 weeks post-PTEN-KO to evaluate for locomotor improvements, totaling 13 weeks post-SCI of survival (see Fig. 1 for timeline).

### Animals and spinal cord injury modeling

All procedures were approved by the University of Kentucky Institutional Animal Care and Use Committee. For experiments that did not require the use of PTEN-KO, female wild-type C57BL/6J mice of age 3 months were used. For experiments that required PTEN-KO, C57BL/6J PTEN-Flox mice were used (B6.129S4-PTEN^tm1hwu^/j; strain #006440; The Jackson Laboratory) as previously described (Stewart et al., 2023). In total, 108 mice were used for all experiments, with 87 mice surviving their respective experimental paradigms for data collection. Attrition was observed due to mortality during or after surgery, bladder rupture, and removal from the study for failed injections or other surgical complications such as torn dura.

Complete spinal crush injuries were performed for all mice in all experiments, using ketamine (100.0 mg/kg) and xylazine (10.0 mg/kg) for anesthesia. A laminectomy was performed at the T9 vertebral level, followed by a complete spinal crush produced using fine-tipped forceps. The forceps were closed around the spinal cord ensuring the tips scraped the ventral side of the spinal column. Force was applied for 8 s before removing the forceps and closing the incision. Mice were placed on a heating pad for recovery. All mice received 1.0 ml of saline support daily for 5 d, as well as enrofloxacin at 5.0 mg/kg/d for 5 d. Buprenorphine’s slow-released formulation (Buprenex SR, 1.0 mg/kg) was provided once after surgery. Daily bladder expressions were provided twice each day until the end of the study.

For delivery of AAVrg's, at 8 weeks post-SCI, mice were again anesthetized as described above and the scar tissue overlying the original laminectomy site was dissected away from the spinal cord. Mice were suspended by vertebral clips and a 10.0-µm-diameter glass-pulled pipette (TIP10TW1-L; World Precision Instruments) was lowered ∼0.5 mm into the center of the spinal cord ∼1.0 mm rostral to the spinal lesion. In many instances, the spinal lesions were unable to be identified, so the needle was placed as close to the T8 vertebra as possible for injections. In total, three mice were confirmed to have injections below the lesions and were not included in the analysis, and one mouse presumably treated with PTEN-KO was removed for having low-to-no visible viral labeling with a concurrent surgical note questioning the success of the injection. One final mouse was removed from the study for possessing an abnormally large and visually identifiable lesion upon re-exposure, of which spinal lesions were not typically observable after re-exposing the cord. A total of 2.0 µl of AAVrg’s (1.0 × 10^9^ gc/µl) carrying either cre-recombinase and dTomato (107738-AAVrg; Addgene), or mCherry alone (114472-AAVrg; Addgene) as a control, was injected into the spinal cord at a rate of 0.3 µl/min. pAAV-hSyn-Cre-P2A-dTomato was a gift from Rylan Larsen (Addgene viral prep #107738-AAVrg; http://n2t.net/addgene:107738; RRID:Addgene_107738). pAAV-hSyn-mCherry was a gift from Karl Deisseroth (Addgene viral prep #114472-AAVrg; http://n2t.net/addgene:114472; RRID:Addgene_114472). The needle was allowed to sit in place for 3 min to restrict backflow through the needle track, and the lesion was closed as described above. Mice received antibiotics, saline, and analgesic support as described above for this second survival surgery.

Upon completion of experiments, mice were killed via an overdose of ketamine (180.0 mg/kg) and xylazine (20.0 mg/kg) followed by cardiac perfusion with PBS and/or 4% formaldehyde prepared from paraformaldehyde powder (1558127; Sigma-Aldrich). For group assignment, animals from all experiments except the PTEN-KO and PLX combinatorial treatment were randomly assigned into groups. For our final PTEN-KO and PLX combinatorial treatment, mice were interspersed into groups at 5 weeks post-SCI prior to beginning treatment with PLX to establish a consistent mean of locomotor recovery [Basso mouse scale (BMS) score] in all groups. Small perturbations to Week 5 mean scores between groups emerged from attrition due to complications arising during the re-exposure surgery for delivering PTEN-KO.

### Transcriptional profiling of the lesioned environment

#### RNA isolation

RNA was isolated for transcriptional analysis using the NanoString Neuropathology panel from either naive mice or at 1, 2, 9, or 12 weeks post-SCI as well as with mice treated with PLX at 7 weeks post-SCI with/without repopulation as described above. Mice were perfused using sterile phosphate-buffered saline (PBS), and ∼5.0 mm of spinal cord encompassing the lesion was isolated and homogenized in RLT lysis buffer containing β-mercaptoethanol (10.0 µl/ml of 14.3 µM β-mercaptoethanol; 63689; Sigma-Aldrich). Debris was pelleted, and the supernatant was passed through genomic DNA eliminator columns prior to purifying on spin columns using the manufacturer’s protocols (RNeasy Plus; 74134; Qiagen). RNA was diluted to 13.0 ng/µl for analysis with NanoString. All RNA was verified for degradation using Bioanalyzer prior to assessments. The NanoString Neuropathology panel was performed at the University of Kentucky Genomics Core Laboratory following the manufacturer’s recommendations.

#### Data analysis

Data were normalized using the NanoString nCounter software, and normalized mRNA counts were obtained. A threshold count of 20 RNA copies was set as a minimal value, which represents approximately twice the value of the geometric mean of the negative controls.

Due to a copious amount of data provided by the NanoString panel across time points and treatment conditions, we have chosen to assess and present data that are directly related to the major hypotheses. Importantly, all data will be made available through the Open Data Commons for Spinal Cord Injury (ODC-SCI; https://odc-sci.org; and all NanoString data will be made available on the Gene Expression Omnibus repository). Relevant to our first objective, data were compared to evaluate for (1) the establishment of a stable inflammatory environment and (2) a deeper understanding of the chronic SCI microenvironment relative to naive controls. To accomplish these tasks, cell-specific transcripts of inflammation were identified and pulled from the data set and evaluated for their levels of expression at all time points. Multiple analysis of variance (MANOVA) was conducted and followed up with Dunnett's pairwise comparison between all other groups and the 12-week timepoint to identify significant changes relative to our last chronic SCI timepoint.

Next, both the 9- and 12-week chronic timepoints were collapsed, and the data were compared with the naive condition to identify differentially regulated genes related to the following cellular compartments including inflammatory cell transcripts, neuron-enriched transcripts, secreted protein transcripts, and extracellular matrix and cell adhesion molecule-related (ECM/CAM) transcripts (see Extended Data Table 1-1 for a list of all assessed genes). All genes used for analysis were Log2-transformed and assessed using multiple *t* tests. The false discovery rate (FDR) was corrected using the Benjamini–Hochberg method.

We hypothesized that depletion would elicit a response from neurons that would be consistent with regeneration or plasticity. To analyze the effects of inflammatory depletion on noninflammatory compartments in the chronic SCI environment, we compared both mice on PLX or mice in the repopulation group to the vehicle-treated chronic SCI controls. mRNA transcripts were pulled and clustered to assess for the inflammatory, neuron-enriched, secreted, and ECM/CAM proteins described above. Each treatment was separately analyzed against the vehicle-treated chronic SCI controls using multiple *t* tests with subsequent FDR corrections. For all NanoString analyses, only genes that possess significant discoveries are presented in figures, and all groups are presented on the same graphs.

### Immunohistochemistry

After fixation with formaldehyde, spinal cords were isolated, acclimated to 30% sucrose solution for 1 week, frozen in blocks of 4–8 cords/block in Shandon M1 Embedding Matrix (1310; Thermo Fisher Scientific), and cut in the sagittal plane at 20.0 µm thickness at −16°C on a cryostat. Tissue sections were picked up directly on slides and labeled for immunohistochemistry. To improve antibody penetration and tissue labeling, particularly in the white matter, all slides were cleared for lipids by passing sections through graded ethanol concentrations to remove water (70, 95, and 100% ethanol), followed by immersing in xylene for 5 min. Slides were subsequently rehydrated by passing back through the ethanol concentrations in reverse order and reacclimating to aqueous PBS. Slides were treated with antigen retrieval consisting of 10.0 mM sodium citrate buffer with 0.05% Tween 20 (pH 6.0) at 80°C for 5 min. The unspecific binding of antibodies was blocked using 5% normal goat serum (NGS) in PBS and 0.1% triton (PBS/T) for 1 h at room temperature. All primary antibodies were incubated at room temperature overnight in PBS/T, and secondary antibodies were incubated for 1 h at room temperature.

To assess β3-tubulin (β3-Tub) axon growth in lesions, one out of every seven sections cut in series were used for analysis. To assess all other outcomes, 1 out of every 14 sections were used for analysis. The percentage of the labeled area within the lesion was used to assess all outcomes and was measured by tracing the GFAP lesion boundaries and setting an intensity threshold on the Indica Labs HALO software (HALO: Indica Labs) to measure positively labeled pixel areas. Total axon labeling was revealed using immunolabeling against β3-tubulin (β3-Tub; 1:2,000; 5568S; Cell Signaling Technology) and was imaged using confocal microscopy. All other outcomes were imaged using conventional fluorescence microscopy on the Axio Scan.Z1 (Carl Zeiss). Total inflammation (Iba-1; 1:4,000; 019-19741; Wako), microglia (P2ry12; 1:2,000; 94555; AnaSpec), serotonin (5-HT; 1:4,000; 20080; ImmunoStar), calcitonin gene–related peptide (CGRP; 1:2,000; 24112; ImmunoStar), and PTEN (1:1,000; 9188; Cell Signaling Technology) were revealed using immunolabeling. Sections were colabeled with astrocytes (GFAP; 1:5,000; Aves Labs) to reveal the lesion boundaries.

### Behavioral monitoring

Mice were monitored for behavioral recovery using the Basso mouse scale (BMS; Basso et al., 2006). Two reviewers who were blinded to experimental groups assessed functional abilities as defined in the BMS scoring systems, allowing at least 4 min per mouse for analysis. In our prior reports, we utilized the Basso, Beattie, and Bresnahan scale of locomotor recovery (Basso et al., 1995) to assess mice with near-complete paralysis due to the increased resolution for lower-limb assessments at the lower ranges of motor abilities. We observed the ability of chronic PTEN-KO to restore weight-supporting abilities in some mice, including weight support in stance, as well as several mice regaining dorsal stepping (Stewart et al., 2023). For this study, we anticipated a similar need to resolve mice transitioning from pre- to postweight supporting abilities and to better depict these improvements in our data. In addition to reporting the BMS scores, we used the predefined scoring criterion on the BMS scale to partition out a subscale that separates out plantar placement without weight support, plantar placement with weight support, and occasional to consistent dorsal stepping, all of which are clustered as a single value of 3 on the BMS. We used this low range subscore to better capture the pre- to postweight supporting transition (Fig. 7).

### Statistical analysis

Statistical analysis of data from the NanoString Neuropathology panel was specific to the objectives; therefore, we applied a priori planned comparisons/analyses. First, we sought to determine the stability of inflammation within the chronic injured environment. Genes attributed to cell-specific inflammatory markers were extracted from the data set and analyzed using a multivariate analysis of variance (MANOVA) with subsequent Dunnett's pairwise comparisons to compare between the naive, 1-, 2-, and 9-week timepoints and the 12-week timepoint (performed in SPSS V28.0.0; all other analyses were performed in Graphpad Prism V10.1.1). We sought to determine the extent to which inflammatory resolution plateaus between the 9- and 12-week timepoints and validated a stable transcriptional environment between 9 and 12 weeks post-SCI. We advanced subsequent analyses using a chronic SCI group that combined both 9- and 12-week timepoints. Next, we wanted to identify significant differentially regulated genes. Individual *t* tests were performed between the combined chronic SCI group and naive controls using the Benjamini–Hochberg method for FDR. All genes deemed significant discoveries were binned into the following categories for presentation: (1) inflammatory cell transcripts, (2) neuron-enriched transcripts, (3) cell-secreted transcripts, and (4) ECM/CAM transcripts (Extended Data Table 1-1). The same analytical strategy was applied to compare chronic SCI to PLX and chronic SCI to mice with inflammatory repopulation.

For immunohistochemistry experiments, when homogeneity of variance demonstrated significant differences between groups using the Brown–Forsythe test, Welch's one-way ANOVA was used with Dunnett's T3 used for pairwise comparisons. Otherwise, one-way ANOVA was used with Dunnett's pairwise comparisons used for post hoc testing. In our combined PTEN-KO and PLX experiments, a second analysis was performed to evaluate the main effects of PLX on axon regeneration by collapsing groups into no-PLX (vehicle and PTEN-KO mice) and PLX-treated mice (PLX and Repop groups). Combined data were assessed using Welch's *t* test. For behavioral assessments, two-way ANOVA with repeated measures was used to compare between groups, followed by the use of Fisher's least significant difference (LSD) for pairwise comparisons. Furthermore, to refine our analysis of behavioral data, we assessed the max performance from all recorded time points pre-PTEN-KO against the max recorded performance post-PTEN-KO using a two-way ANOVA with post hoc assessment using Fisher's LSD to compare within-group and vehicle-treated conditions.

## Results

### Chronic SCI lesions are hallmarked by a sustained elevation of inflammation with a persistent differential regulation in the secretome and extracellular matrix

To determine the role of chronic intraspinal macrophage activation on axon growth and regeneration, we first sought to identify a chronic timepoint in our model with nonresolving, stable, and persistent inflammation. Specifically, we utilized the NanoString Neuropathology panel to quantify mRNA transcripts related to inflammation and neuropathology in tissue isolated from uninjured (naive) spinal cords or spinal cords at 1, 2, 9, and 12 weeks after severe thoracic T9 crush SCI (Fig. 1*A*). We defined chronicity as no significant change of inflammation between two identified timepoints.

**Figure 2.**
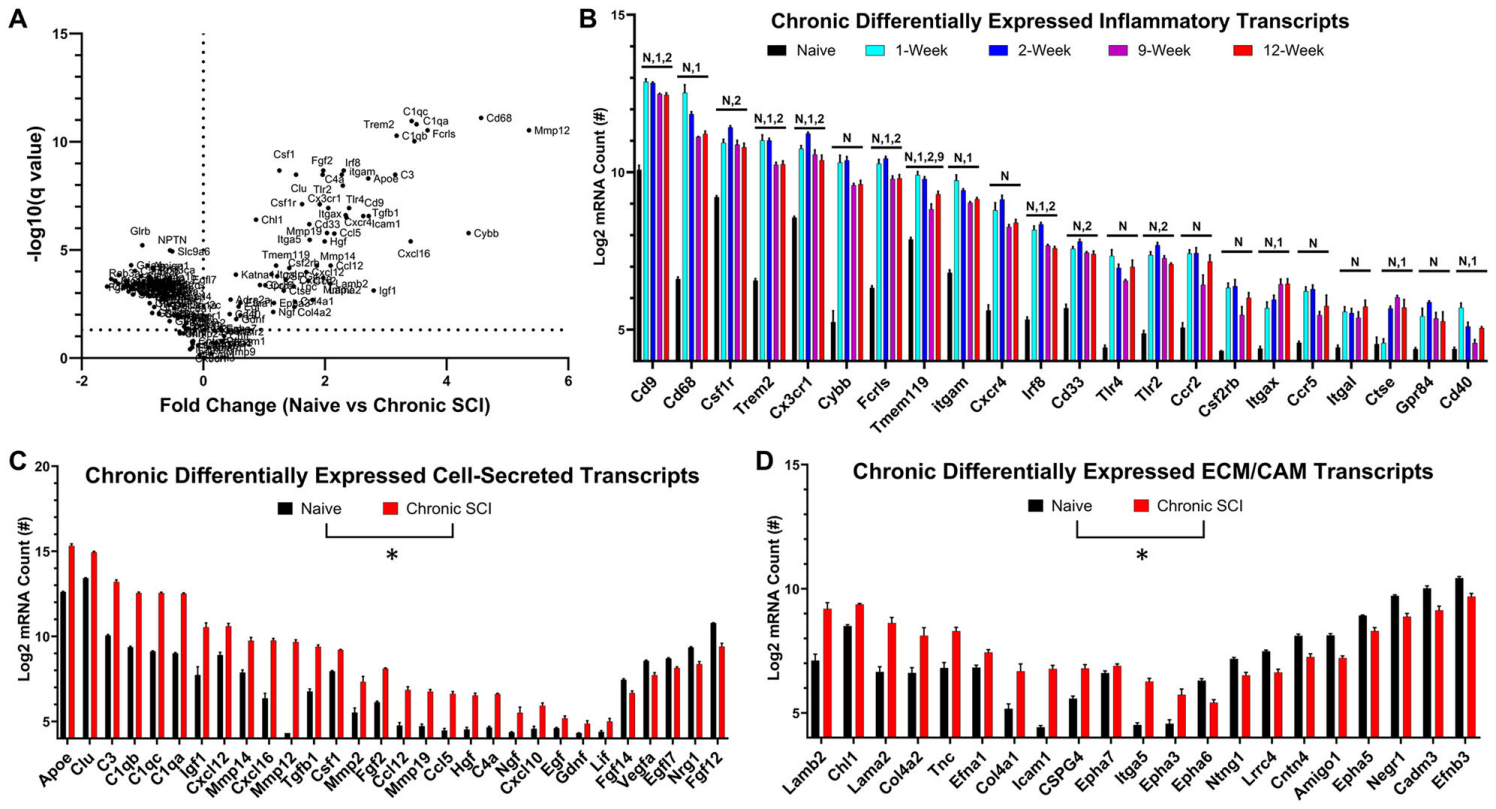
Nonresolving inflammation is a hallmark of chronic SCI and retains a lesion microenvironment with differentially regulated cell-secreted and extracellular matrix transcripts. Transcriptional analysis (***A***) of the lesioned microenvironment reveals peak inflammatory profiles between 1 and 2 weeks post-SCI with incomplete resolution toward uninjured levels that plateaus between 9 and 12 weeks postinjury (***B***). Comparing naive to chronic SCI lesions displays a microenvironment with persistent differential regulation of the extracellular compartment including cell-secreted (***C***) and extracellular matrix and cell adhesion molecule (ECM/CAM)-related transcripts (***D***). Transcripts displaying a persistent upregulation in chronic SCI tend to be inflammatory-related, while genes downregulated in chronic SCI tend to be transcripts that are enriched in neurons, with diverse effects on cell-secreted and extracellular matrix transcripts (***A***; Extended Data Table 1-1). Error bars = SEM. *p* < 0.05 for 12 weeks vs N, naive, 1 = 1 week, 2 = 2 weeks, and 9 = 9 weeks post-SCI using MANOVA and Dunnett's pairwise comparisons (***B***). *p < 0.05 after FDR correction for all genes displayed (***C***, ***D***).

Similar to previous reports (Beck et al., 2010; Kwiecien et al., 2020), we observed that inflammatory markers peaked between 1 and 2 weeks post-SCI, with a decrease to stable expression observed between 9 and 12 weeks post-SCI (Fig. 2). Only 1 out of 23 of our assessed inflammatory markers, TMEM119, changed between the 9- and 12-week timepoints (*p* = 0.039), and expression increased rather than decreased (Fig. 2). Moving forward, we collapsed the 9- and 12-week timepoints into a single chronic SCI group for comparisons.

10.1523/JNEUROSCI.1017-24.2024.t1-1 Table 1-1 Gene names, *p* values, means, standard deviations, and sample sizes for transcripts used from the Nanostring Neuropathology panel in Figuers 2-5. *Note: NA = RNA counts too low to assess, not included in the analysis. Naive vs Chronic SCI was assessed in a separate batch of Nanostring assessments from the Chronic SCI vs PLX and Repop groups. Chronic SCI mice from the PLX-depletion experiments were assessed with PLX and Repop groups on a separate Nanostring panel together at a different time. Variability between Chronic SCI conditions is due to performing the experiments at different times and running on Nanostring in different batches/cohorts. Comparisons of mRNA counts should only be performed between other groups within their respective experiments. All mean mRNA counts are provided prior to Log2 transformation. *p* = results from T-Tests before FDR correction. *q* = corrected p value after FDR correction. Download Table 1-1, XLS file.

Of the 162 selected mRNA transcripts examined between naive and chronic SCI mice, 140 transcripts were significantly different after FDR correction. Specifically, 23/23 inflammatory, 65/66 neuron-enriched, 29/37 secreted, and 21/34 ECM/CAM mRNA transcripts were differentially regulated between naive and chronic SCI conditions. Collectively, transcriptional analysis of tissue around chronic SCI lesions supports a persistent change in the microenvironment that may play both a role in affecting local neuronal physiology as well as affecting the potential for axon growth and repair (Extended Data Table 1-1).

### CSF1R antagonism with PLX-5622 in chronic SCI depletes macrophages and microglia both within and surrounding the lesion

While we confirmed that persistent inflammation remains a hallmark of chronic SCI and stabilizes between 2 and 3 months after SCI, the influence of sustained inflammation on ongoing functions and/or tissue repair remains to be fully elucidated. To determine the role of chronic intraspinal inflammation on repair processes, we first depleted microglial and macrophages through CSF1R antagonism. Treatment with the CSF1R antagonist, PLX-5622 (PLX), was initiated at 11 weeks postinjury and maintained until spinal cord tissue was harvested either 9, 14, or 21 d later (Fig. 1*B*). One additional group was allowed to recover for 21 d after removal of PLX before tissue isolation at 17 weeks post-SCI (Fig. 1*B*).

**Figure 3.**
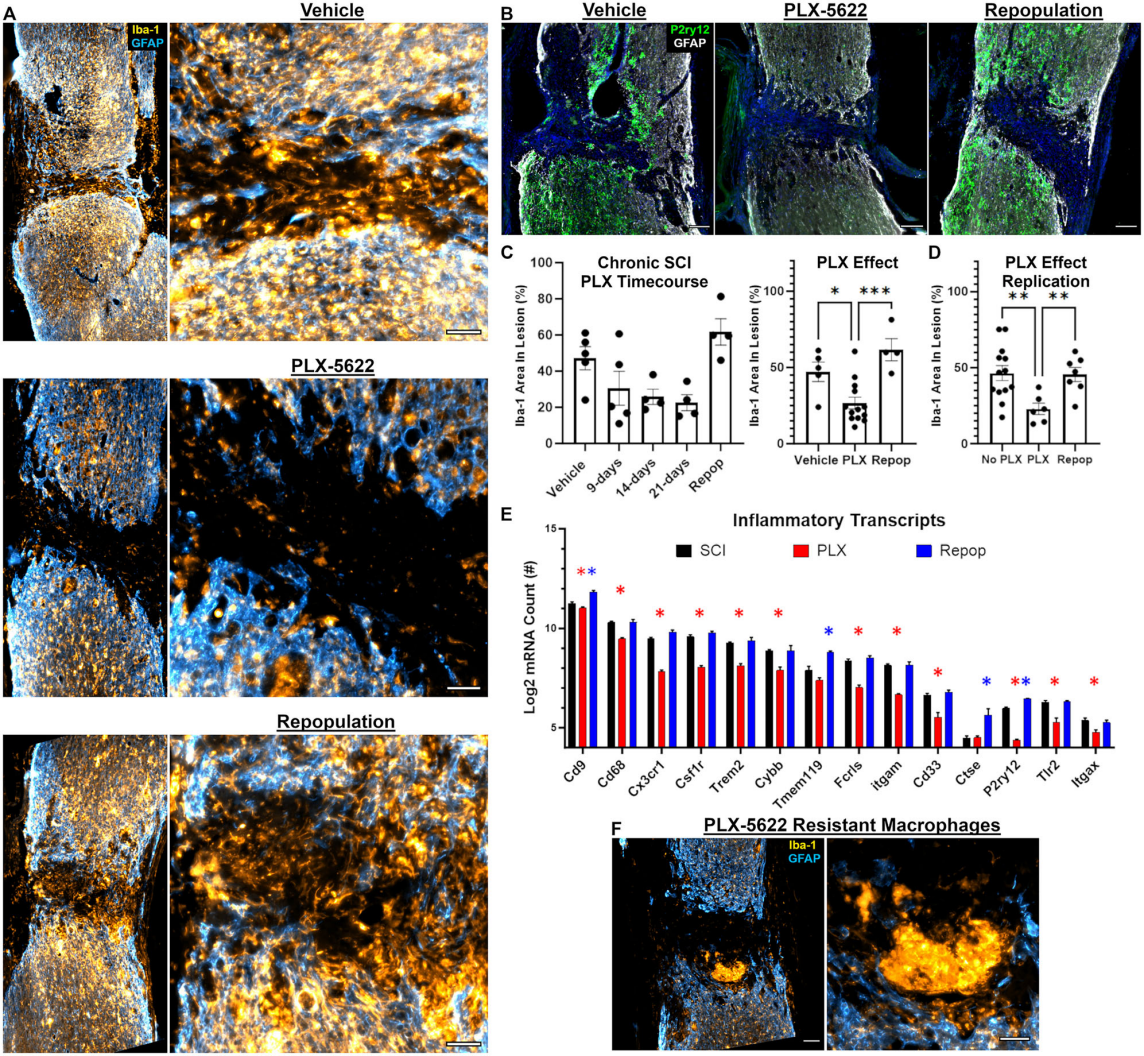
Macrophages and microglia repopulate the lesion environment back to predepleted levels upon removal of PLX-5622 treatment when delivered in chronic SCI. Representative images of the site of SCI after treatment of vehicle, PLX-5622, or after removal of PLX-5622 (repopulation, see Fig. 1*B* for timeline; ***A***, ***B***). CSF1R antagonism with PLX-5622 delivered at 11 weeks (***C***), or 6 weeks (***D***), post-SCI depletes Iba-1^+^ macrophages and microglia from within the lesion boundaries (***A***, ***C***). Macrophages and microglia repopulate the lesioned environment within 21 d of removing PLX-5622 (***A***, ***C***, ***D***). The microglial-specific marker, P2ry12, continues to display perilesional expression and low-to-no expression within the lesion core in chronic SCI (***B***). Repopulating macrophages and microglia that reinfiltrate the lesion does not express microglial-specific P2ry12 (***B***). Transcriptional analysis of the lesioned environment demonstrates consistent findings with immunohistochemistry (***E***). Specifically, PLX-5622 depletes microglia/macrophages across multiple inflammatory-related markers, but the removal of treatment restores inflammation back to, or above, predepleted levels (***E***; Extended Data Table 1-1). While PLX-5622 does significantly deplete inflammation within the lesion, some Iba-1^+^ cells display resistance to PLX-5622 and are retained during treatment (***F***). Effects of PLX-5622 on inflammatory depletion and repopulation within the lesion were replicated in a second cohort of mice from experiments described in the section “Macrophage/microglial depletion and repopulation exert fiber-type–specific axon regeneration into the lesion which is independent of PTEN-KO” (no-PLX group combined both vehicle-treated and PTEN-KO/only mice; ***D***). Scale bars: 100 µm (***B***, ***F*** left), 50 µm (***A***, ***F*** right). Error bars = SEM. *p < 0.05, **p < 0.01, ***p < 0.001 (***C***, ***D***, ***E***). *p < 0.05 after FDR correction (***E***).

Histological evaluation of microglial (P2ry12) and pan macrophage/microglial (Iba-1) markers revealed significant reductions in macrophages/microglia with PLX treatment that were evident by 9 d posttreatment, with stable depletion by 14 d, and no further decrease in macrophage/microglial markers between 14 and 21 d posttreatment (Fig. 3). Of important note, not all Iba-1^+^ cells were depleted from within the lesion. Larger and more round-in-appearance macrophages within the lesion did not appear to deplete with PLX treatment (Fig. 3*F*). When PLX treatment was discontinued for 3 weeks, Iba1^+^ cells repopulated the lesion site to the same extent as vehicle-treated controls (Fig. 3*A,B*). For statistical comparison, we collapsed the PLX-treated groups to compare the effects of PLX on vehicle-treated mice and mice allowed for inflammatory repopulation. PLX exerted a significant depletion effect (*F*_(2,19)_ = 10.87, *p* = 0.0007) between vehicle-treated (*p* = 0.024) and repopulation mice (*p* = 0.0007; Fig. 3). These effects were independently verified in a second cohort of animals from mice described in the section “Macrophage/microglial depletion and repopulation exert fiber-type–specific axon regeneration into the lesion which is independent of PTEN-KO” below (Fig. 3*D*). Specifically, we observed a depletion effect (*F*_(2,23)_ = 5.58, *p* = 0.01) between mice treated with PLX and those that did not receive PLX (combined vehicle controls and PTEN-KO–treated mice; *p* = 0.007) as well as between mice allowed for 21 d of inflammatory repopulation (*p* = 0.022; Fig. 3*D*).

As mentioned above, upon removal of PLX, macrophages and microglia repopulated back to predepleted levels by 21 d after treatment removal. We labeled tissue sections for the microglial protein, P2ry12, to determine if inflammation within the lesion was microglia in origin. As previously reported, macrophages within the lesion prior to depletion largely do not express P2ry12 (Stewart et al., 2021). In our study, Iba-1^+^ cells that repopulated the lesion similarly do not express P2ry12, while repopulation at the lesion penumbra does express P2ry12 (Fig. 3*B*). While our data do not support the repopulation of microglia into the lesioned environment, we similarly cannot conclude that repopulating macrophages within the lesion are not derived from microglia in origin. Further work should identify the source of repopulating inflammation in the different compartments within the injured spinal cord (e.g., within the lesion vs penumbra).

To better characterize the inflammatory depletion and repopulation observed by histology, we replicated the depletion experiments and focused on isolating tissue 9–12 weeks postinjury to encompass stable nonresolving intraspinal inflammation (Fig. 2). As outlined in Figure 1, we treated SCI animals with PLX for 2 weeks, starting treatment at 7 weeks postinjury. Spinal cord homogenates encompassing the lesion were then isolated during depletion (9 weeks postinjury) or after repopulation (12 weeks postinjury) and analyzed using the NanoString Neuropathology panel for transcriptional analysis of mRNA. A control cohort received normal chow, i.e., SCI vehicle control, with half the cohort killed at 9 weeks and the other at 12 weeks postinjury to control for time post-SCI. Consistent with our histology, we observed a significant decrease in 14/23 markers enriched in macrophages and microglia in the depleted group relative to SCI controls (Fig. 3*E*). After PLX was discontinued, all 14 transcripts returned to nontreated SCI values with two markers specific for microglia, P2ry12 (*q* = 0.002) and TMEM119 (*q* = 0.03) displaying a significant elevation above untreated levels after repopulation (Fig. 3*D*).

Collectively, between three separate replications and across two separate outcomes, we observed the ability of PLX to deplete inflammation both within and surrounding chronic SCI lesions; however, upon removal of treatment, macrophages and microglia repopulate back into the lesion up to, or above, predepleted levels. Of important note, not all Iba-1^+^ cells were depleted from within the lesion. Larger and more round-in-appearance macrophages within the lesion either did not deplete with PLX treatment or were recruited post-PLX delivery to clear apoptotic cells (Fig. 3*F*).

### Inflammatory repopulation elicits global changes to the tissue microenvironment

**Figure 4.**
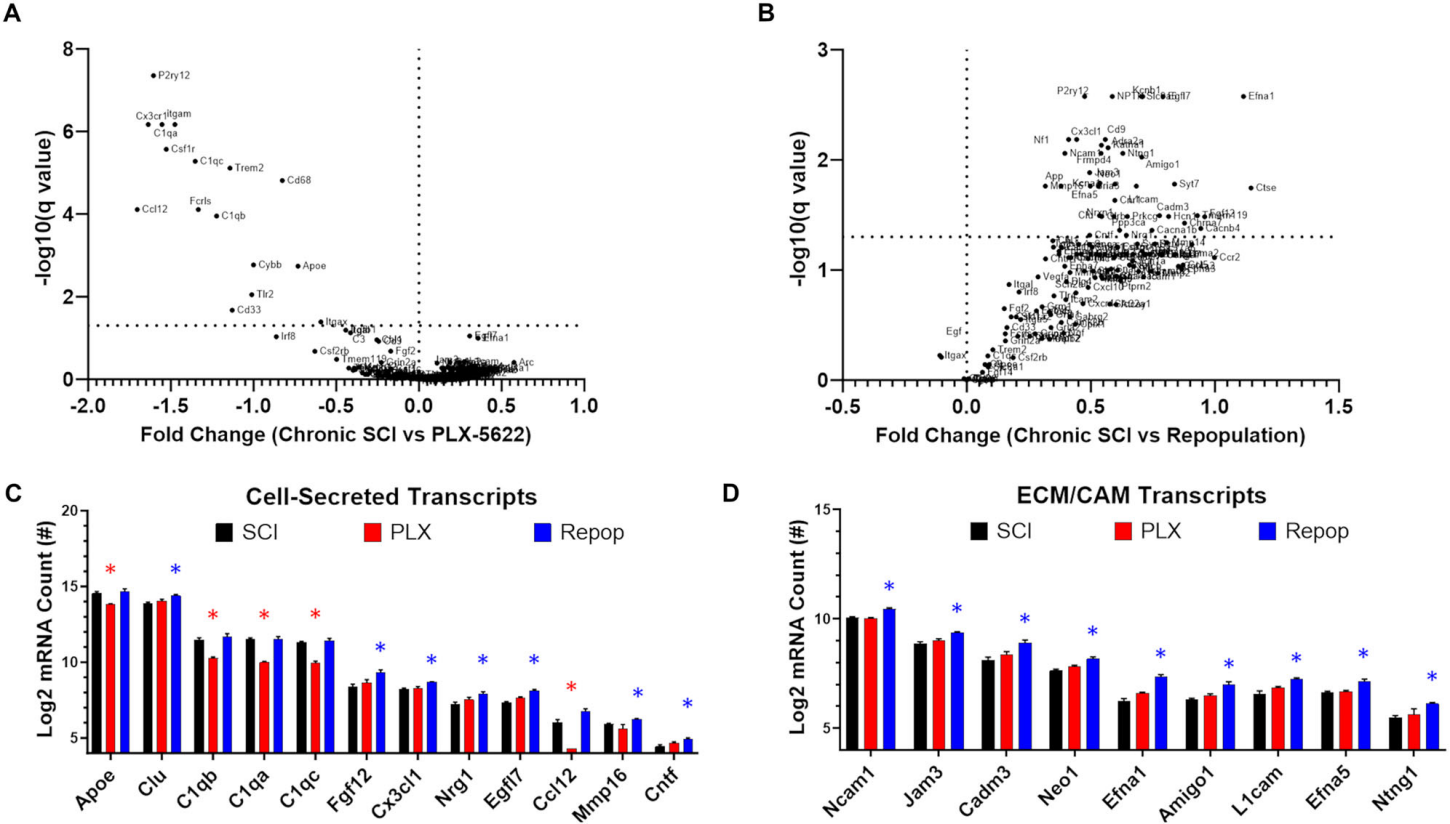
Inflammatory repopulation after PLX-5622 removal, but not depletion alone, influences the extracellular environment in chronic SCI. Delivery of PLX-5622 for 14 d starting at 7 weeks post-SCI exerts minimal effects on the extracellular environment (***A***) beyond depleting transcripts enriched in macrophages and microglia (***C***, ***D***). Inflammatory repopulation at 21 d after treatment removal (see Fig. 1*C* for experimental design) exerted significant changes to the extracellular environment (***B***) including an upregulation of several cell-secreted and extracellular matrix and cell adhesion molecule (ECM/CAM) transcripts (***C***, ***D***). Transcripts that were depleted by PLX-5622 were restored to predepleted values with the re-emergence of macrophages and microglia within the spinal cord (***C***; Extended Data Table 1-1). Error bars = SEM. *p < 0.05 versus SCI controls after FDR correction (***C***, ***D***).

We next examined the effect of macrophage/microglial depletion and repopulation on cell-secreted, extracellular matrix, and neuron-enriched transcripts. We hypothesized that the elimination of macrophages and microglia would allow for a reparative response of neurons and other tissues around the lesion. Contrary to our hypothesis, we did not observe a significant change in the microenvironment through NanoString transcriptional analysis after 2 weeks of inflammatory depletion (Fig. 4*A*). The only significantly affected transcripts during inflammatory depletion, compared with untreated SCI controls, were reduced inflammatory markers and closely corresponding genes that are known to be highly enriched in macrophages and microglia (e.g., complement proteins; Fig. 4*C*). There were no significant differences with depletion on ECM/CAM transcripts relative to untreated SCI controls (Fig. 4*D*). In contrast, repopulation was associated with a significant upregulation of many noninflammatory related transcripts relative to nontreated SCI controls (Fig. 4*B*). Specifically, we observed significant increases in transcripts of secreted molecules (7/37) associated with cell migration and growth, e.g., fibroblast growth factor (Fgf12), ciliary neurotrophic factor (Cntf), and neuroregulin (Nrg1), as well as ECM/CAM-related transcripts (9/34), e.g., neural cell adhesion molecule 1 (Ncam1), junctional adhesion molecule 3 (Jam3), and cell adhesion molecule 3 (Cadm3; Fig. 4).

**Figure 5.**
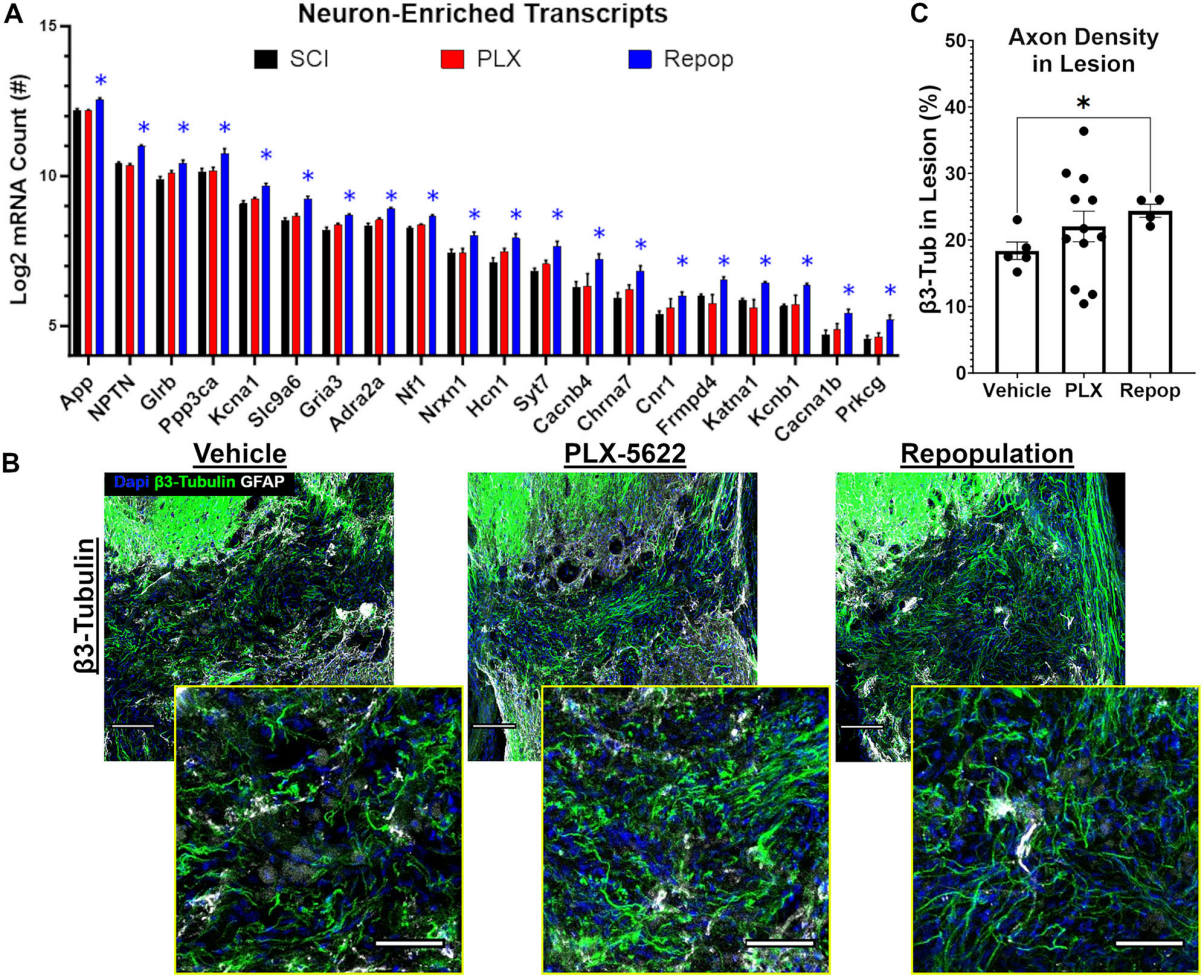
Inflammatory repopulation after PLX-5622 removal upregulates neuron-enriched transcripts within the injured environment and increases axon densities within the lesion. Lesions of mice treated with PLX-5622 and allowed for inflammatory repopulation displayed a significant upregulation of neuron-enriched transcripts (***A***; Extended Data Table 1-1). Correspondingly, immunohistochemical analysis displayed a significant increase in axon densities within the lesions of mice allowing for inflammatory repopulation, with trends toward an increase with depletion alone (***B***, ***C***). Importantly, mice treated with PLX-5622 were allowed to survive for 9–21 d posttreatment initiation, while repopulated lesions were assessed 42 d posttreatment initiation and 21 d posttreatment removal. For immunohistochemical analyses, one mouse was killed at each timepoint to control for total time post-SCI. For transcriptional analyses, three SCI-control mice were killed alongside mice which received 14 d of PLX-5622, and three mice were killed alongside mice that were allowed 21 of repopulation after treatment removal. Scale bars: 100 µm for less magnified images and 50 µm for zoomed images. Error bars = SEM. *p < 0.05 after FDR correction versus SCI controls (***A***). *p < 0.05 (***C***).

Given the well-established interplay among inflammatory cells, secreted growth factors, and ECM/CAM proteins with neuron survival and axon growth, we next examined the effects of inflammatory depletion/repopulation on transcripts that are enriched in neurons. Of important note, while many of our selected transcripts have a high expression in neurons, several are concurrently expressed in other cell types. Depletion alone did not significantly alter neuron-enriched transcripts relative to nontreated SCI controls (0/66; Fig. 5*A*). In contrast, several neuron-enriched transcripts were significantly increased after inflammatory repopulation relative to nontreated SCI controls (20/66). Collectively, transcriptional analysis identified that the chronic SCI environment differs depending on inflammatory depletion and/or repopulation, with each treatment condition presenting a unique environment that confers a potential influence over axon growth and regeneration. Maintaining mice on PLX depletes inflammatory cells and eliminates their contributions to the microenvironment, while repopulation of inflammation elicits a significant transcriptional response from other cells, potentially including neurons, as well as affects several ECM/CAM-related transcripts known to play a role in axon guidance (Fig. 4).

### Repopulation of macrophages elicits a neuronal response that augments axon densities in chronic SCI lesions

Having observed that macrophage depletion and repopulation significantly influence the chronic SCI environment, we sought to determine if either condition alone would augment axon growth into the lesion using histological analyses. Specifically, we labeled for the pan-neuron marker, β3-tubulin, and assessed axon densities within the lesion. We observed a significant increase in axon densities (*W*_(2,10.74)_ = 6.372, *p* = 0.015) in chronic SCI lesions in inflammatory repopulated mice (*p* = 0.015). While we did not detect a significant increase with depletion alone (*p* = 0.33), the range of axon densities greatly exceeded even the lesions with inflammatory repopulation (Fig. 5*B,C*), indicating some prospective influence of axons with depletion alone.

### Macrophage/microglial depletion and repopulation exert fiber-type–specific axon regeneration into the lesion which is independent of PTEN-KO

Our primary goal was to determine the role of sustained inflammation on axon regeneration in chronically injured spinal cord. Endogenous regeneration and repair are limited after SCI. We therefore employed a combinatorial treatment using both PTEN-KO, a well-established proregenerative treatment to drive axon regeneration (Liu et al., 2010; Danilov and Steward, 2015; Du et al., 2015; Geoffroy et al., 2015), and PLX, in chronic SCI. As illustrated in Figure 1*D*, we initiated PLX treatment 6 weeks after SCI and PTEN-KO 8 weeks after injury. Tissue was then harvested at a timepoint indicative of chronic, sustained intraspinal inflammation (13 weeks postinjury). We included both a group with mice sustained on PLX for 7 weeks throughout PTEN-KO and a group treated with PLX for only 4 weeks which allowed for 21 d of inflammatory repopulation in the presence of PTEN-KO. We used spinal injections of retrogradely transported AAVs (AAVrg's) as previously described (Metcalfe and Steward, 2023; Stewart et al., 2023) to knock-out PTEN as a growth-promoting stimulus that targets most spinal-projecting neurons descending from the brain and brainstem. Control groups included animals without PLX or PTEN-KO (aka vehicle SCI) and animals treated for PTEN-KO without macrophage depletion (PTEN-KO alone).

**Figure 6.**
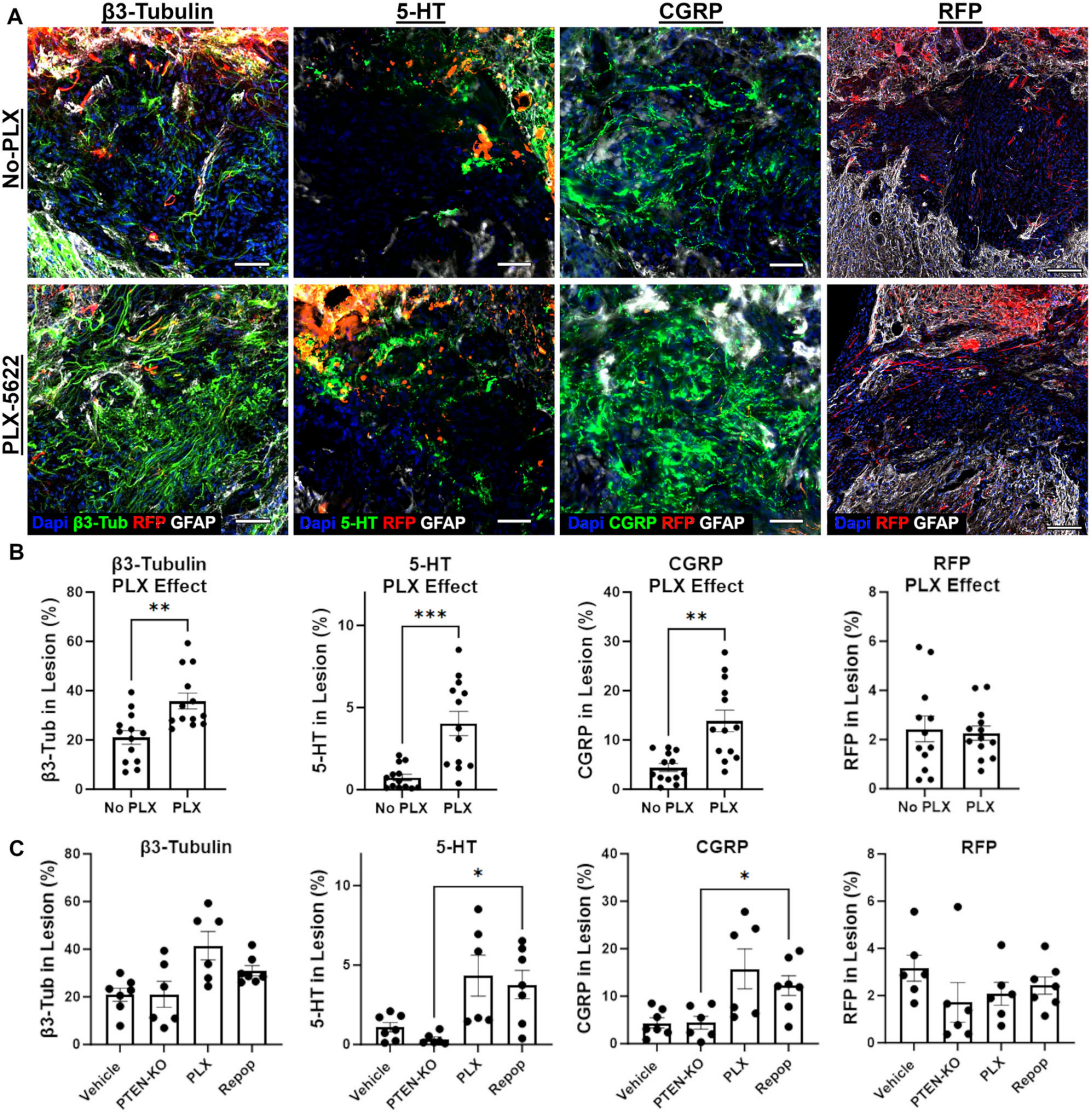
5-HT^+^ and CGRP^+^ axons expand within PLX-5622 treated chronic SCI lesions, but PTEN-KO with AAVrg's does not further augment axon growth into the lesion. Mice were treated for 4 (Repop group; 4 weeks of treatment 3 weeks of treatment recovery) or 7 weeks (PLX group) with PLX-5622 or a regular diet as a vehicle control, beginning at 6 weeks post-SCI. Mice were subsequently subject to PTEN-KO or a vector control using AAVrg's to deliver cre-recombinase and RFP or RFP alone at 8 weeks post-SCI (2 weeks post-PLX-5622 initiation; Fig. 1*D*). Total axon growth increased in the lesions of mice treated with PLX-5622 regardless of allowing for inflammatory repopulation (***A***, ***B***). No significant increase in RFP^+^ axon growth was observed in the lesions of any PTEN-KO group relative to vehicle controls, indicating that PLX-5622 does not augment the regeneration of axons susceptible to AAVrg's (***A–C***). Both 5-HT^+^ and CGRP^+^ axon densities were significantly increased within chronic SCI lesions in response to PLX treatment, independent of inflammatory repopulation (***A***, ***B***). A significant increase was observed between the repopulation group and PTEN-KO controls at the level of individual comparisons (***C***). Scale bar: 50 µm. Error bars = SEM. *p < 0.05, **p < 0.01, ***p < 0.001.

To first evaluate overall axon growth, regardless of phenotype, we labeled for β3-tubulin to visualize all axons. We replicated the main effect of PLX on axon growth within the lesion when collapsing groups into those that either did or did not receive PLX (*p* = 0.002; Fig. 6*B*). When assessing all four groups individually, we observed a significant main effect of treatment (*W*_(3,10.97)_ = 4.544; *p* = 0.026) with the greatest difference between PTEN-KO alone versus PTEN-KO with PLX administration with or without repopulation (Fig. 6*C*).

Observing an increase in axons within the lesions of mice treated with PLX replicated our prior experiment, however, here we demonstrate that axon growth is independent of sustained depletion or repopulation when total time postdepletion was controlled (Fig. 6*B*). Next, we investigated axon growth of specific populations including serotonin expression axons (5-HT) and calcitonin gene–related peptide (CGRP)-positive axons which are not efficiently transduced with AAVrg's. We detected a significant main effect of PLX treatment with increased axon growth of both 5-HT (*p* = 0.007) and CGRP (*p* = 0.001) when collapsing groups into those that did or did not receive PLX (Fig. 6*B*). By comparing PTEN-KO–treated mice to all other groups, we identified a significant increase in 5-HT^+^ (*W*_(3.0,10.48)_ = 8.01, *p* = 0.0046) axon densities within the lesions of mice allowed for inflammatory repopulation (*p* = 0.023), but not in mice with sustained inflammatory depletion (*p* = 0.07; Fig. 6*C*). Similar observations were made when evaluating fibers histologically identified as CGRP^+^ (*W*_(3.0,11.23)_ = 5.34, *p* = 0.015), with a significant increase in axon densities observed between PTEN-KO and inflammatory repopulation conditions (*p* = 0.031), but not with depletion alone (*p* = 0.1; Fig. 6*C*). Interestingly, neither depletion (*p* = 0.97) nor repopulation (*p* = 0.81) affected RFP^+^ axon growth into the chronic lesioned environment beyond mice treated with PTEN-KO (Fig. 6), indicating that PLX may augment growth of specific axon subtypes that possess pre-existing abilities for limited growth into the lesion (Inman and Steward, 2003).

Collectively, we have reproduced an effect of PLX on axon growth into chronic SCI lesions with both sustained and repopulated inflammatory conditions; however, macrophage repopulation elicits a more consistent axon growth response. While we did not observe a significant augmentation of retrogradely labeled RFP^+^ fibers, this does not preclude the possibility that axons transduced by AAVrg's could have grown around the lesion and may not be capable of intralesion growth, despite inflammatory depletion. Here, we identify at least two histologically identifiable axons with some potential for growth into the lesion that respond to macrophage/microglial depletion with significant growth within the lesion.

### Inflammatory repopulation with PTEN-KO improves locomotor functions

**Figure 7.**
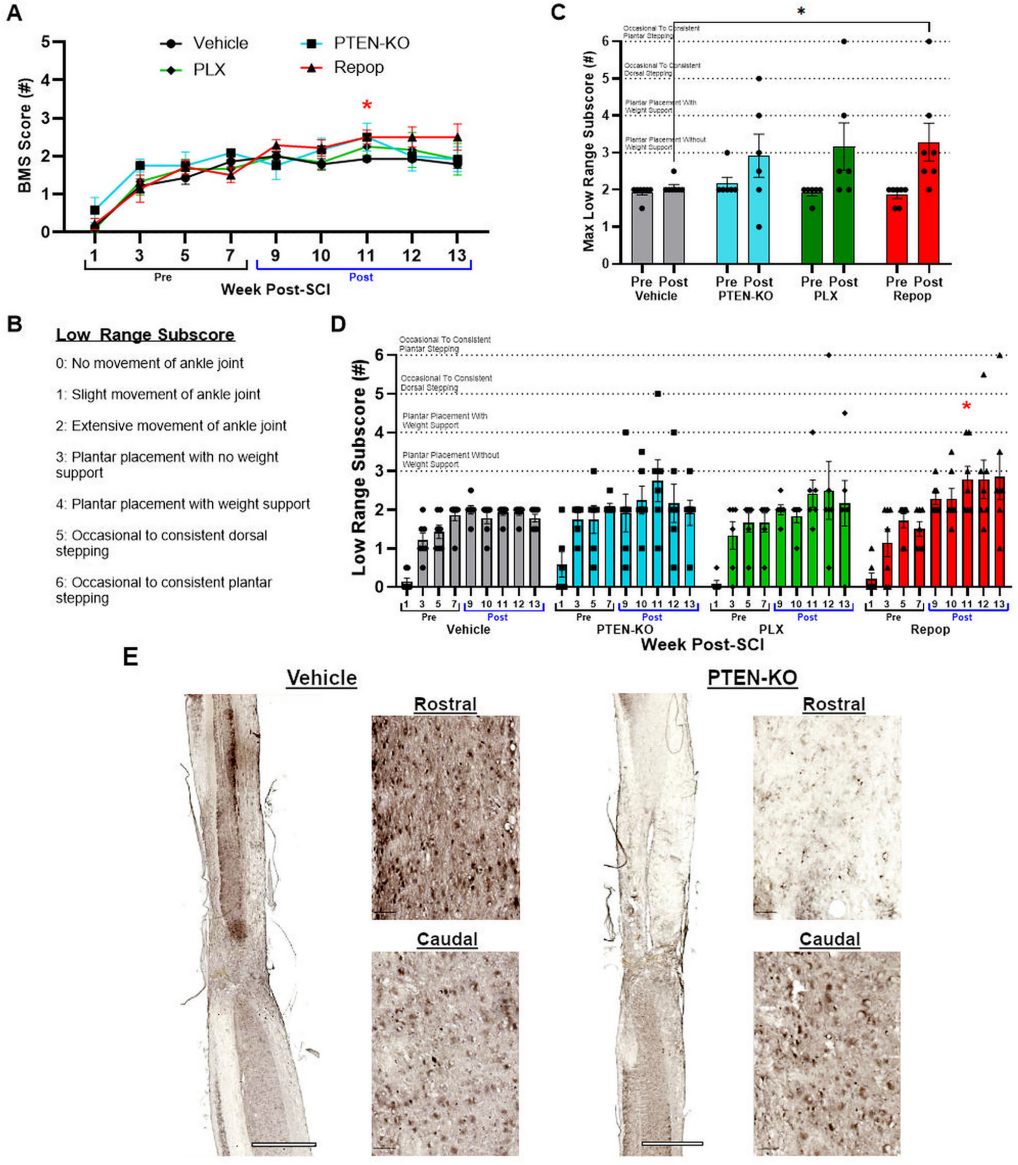
PTEN-KO with AAVrg's restores weight support in chronic SCI, but PLX-5622 does not significantly affect locomotor functions. Mice were treated for 4 (Repop group; 4 weeks of treatment, 3 weeks of treatment recovery) or 7 weeks (PLX group) with PLX-5622 or a regular diet as vehicle control, beginning at 6 weeks post-SCI. Mice were subsequently subject to PTEN-KO or vector control using AAVrg's to deliver cre-recombinase and RFP or RFP alone at 8 weeks post-SCI, respectively (2 weeks post-PLX-5622 initiation; Fig. 1*D*). No main effects were found using two-way ANOVA for the BMS (***A***), despite observing 6/19 mice treated with PTEN-KO regaining weight support of some kind after treatment (***C***, ***D***). A low range subscore was generated to represent mice transitioning from pre- to postweight supporting abilities using predefined BMS criterion (***B***). When observing the maximal recorded performance from all pre- and post-PTEN-KO mice, within-group improvements were detected for both mice sustained on PLX and allowed for repopulation, but no differences were observed above PTEN-KO only (***C***). Immunohistochemistry against PTEN was performed to validate knock-out at the injection location (***E***). Larger neuronal cell bodies were not stained for PTEN at the injection location, while labeling was still apparent below the lesion as well as at the injection site in control-treated mice (***E***). Error bars = SEM. *p < 0.05, **p < 0.01, ***p < 0.001.

We monitored for potential synergistic effects on locomotor recovery derived from combining PTEN-KO and PLX with and without repopulation. As a main effect over the duration of the study, we did not detect significant between-group differences (*F*_(3,22)_ = 0.893, *p* = 0.46), nor a significant time-by-group interaction (*F*_(24,176)_ = 1.124, *p* = 0.32). However, at the level of pairwise comparisons, we did observe significant differences between the inflammatory repopulation group and vehicle controls at 3 weeks posttreatment (*p* = 0.023) using the BMS as a main outcome (Fig. 7*A*). Not observing a significant improvement as a main effect on the BMS was surprising considering we observed several mice in all groups receiving PTEN-KO regain weight-supporting abilities at some point after treatment (6/19 mice treated with PTEN-KO with or without PLX).

To gain better resolution that can capture the pre- to postweight supporting hindlimb abilities, we generated a low range subscale based on the BMS scoring criterion (Fig. 7*B*). Prior to treatment, only one animal across all groups demonstrated hindlimb function better than extensive ankle movement (plantar placement of the hindlimb, PTEN-KO–alone group). After intervention, there were no significant improvements in the vehicle-only group with only two animals (2/7, 29%) showing any increased function at any time after treatment (*p* = 0.75; Fig. 7). Although more animals recovered function in the PTEN-KO–alone group (3/6, 50%), the maximal performance before versus after treatment was not statistically significant (*p* = 0.14). In contrast, in both the macrophage-depleted and macrophage-repopulated groups, significant improvements were detected as a maximal recorded performance pre- versus posttreatment (*p* = 0.02 and *p* = 0.005, respectively; Fig. 7*B*). Specifically, five of six animals in the PLX-treated group improved with treatment (83%) and seven of seven in the depletion and repopulation groups (100%). Relative to vehicle control–treated animals, only the repopulation group differed between vehicle controls at 11 weeks posttreatment (*p* = 0.046), as well as in their max performance posttreatment (*p* = 0.039; Fig. 7*B*). While our data implicate most of the treatment effect is likely derived from PTEN-KO, mice receiving both PTEN-KO with inflammatory repopulation displayed the largest effect that was significantly different between vehicle-treated controls.

## Discussion

Persistent intraspinal inflammation after SCI has been documented across most mammalian species including humans (Beck et al., 2010; Kwiecien et al., 2020; Zrzavy et al., 2021); however, little is known about the role of chronic inflammation after SCI. At least two potential hypotheses exist that can explain the persistence of increased inflammation after SCI: (1) macrophages within the lesion core become trapped and undergo phenotypic changes throughout the progression of SCI pathology, or (2) macrophages within the lesion undergo life cycles of death and repopulation at a rate which perpetuates sustained inflammation. The first hypothesis would be supported by an environment rich in ECM/CAM that prohibits growth and migration of tissue, as well as a well-characterized transition of macrophages toward a foam-cell phenotype that restricts cellular migration (Luo et al., 2017; Zhu et al., 2017).

After significantly depleting inflammatory populations from the lesion core, inflammation repopulated back to elevated and predepleted levels. While repopulation of tissue-resident macrophages has already been described after removal of PLX in other nervous system and systemic tissues (Henry et al., 2020; Lei et al., 2020), we speculated that the extent of repopulation would approach levels closer to naive conditions. Observing a repopulation back to predepleted, and hyperinflated, levels supports the role of the chronic SCI environment in sustaining elevated macrophage/microglial densities around the lesion as a new homeostasis, potentially through ongoing turnover within the lesioned environment.

While we did label against P2ry12 in attempts to identify the source of depleting/repopulating cells within the lesion, we cannot make strong conclusions about the origins of depleted cells within the lesion being either macrophage or microglia in nature. While several studies have applied similar CSF1R antagonism approaches in pre-SCI or acute SCI conditions, only a modest depletion of macrophages has been reported with a strong preference for microglia (Bellver-Landete et al., 2019; Brennan et al., 2022). We are unaware of any experiments that applied CSF1R antagonism in chronic SCI and evaluated the cell-specific depletion effects. It is likely that our experiments similarly exerted a microglial preference for depletion; however, the identity of lesion-dwelling Iba1^+^ cells was not determined in our experiments. There remains a need to further understand nonresolving neuroinflammation during the late stages of chronic SCI.

Transcriptional analysis of the chronic SCI environment revealed a significant and persistent elevation of CSF1 (Fig. 2*C*), which was not significantly affected by macrophage depletion (Extended Data Table 1-1). A chronic elevation of CSF1 could explain an increased support for macrophage/microglia within and surrounding the lesion. We originally hypothesized that a persistent increase in inflammation was supporting chronic activation of the glial scar and a persistent expression of inhibitory ECM/CAM such as NG2/CSPG4. However, our data point to the contrary, specifically supporting that noninflammatory cells around the lesion sustain increased densities of macrophages and/or microglia within the SCI environment. Transcriptional analysis after inflammatory depletion did not reveal a significant effect on any cellular marker that could be attributed to noninflammatory components of the lesioned environment. Instead, we observed a decrease in macrophage-/microglial-specific markers and their known contributions to the secretome such as complement proteins. Importantly, one recently published paper has suggested that delivery and sustained CSF1R antagonists in chronic SCI for 10 weeks can resolve astrogliosis (Xia et al., 2022). While our data do not replicate such a conclusion, our transcriptional experiments only sustained mice for 2 weeks, which may provide insufficient time for the glial scar to resolve. It remains possible that sustaining longer depletion times or using more sensitive outcome measures specifically targeting gliosis or other cellular responses may yield different conclusions.

Observing that inflammatory repopulation elicited an upregulation of neuronal-enriched genes implicates a role for proinflammatory events in chronic SCI to regulate neural responses that may include repair and regeneration. The reason for a global response to repopulation remains unknown; however, we speculate that an environment filled with proliferating cells may increase the secretion of growth/trophic factors which would interact with other cells in the environment. The role of inflammatory events in the CNS during chronic stages of SCI has been previously explored for the ability to promote periods of plasticity that can be fostered for functional recovery but the underlying mechanisms remain to be elucidated (Torres-Espín et al., 2018).

Of potential importance was a significant upregulation of MMP16 and a notable but insignificant upregulation of MMP14 (*p* = 0.058 after FDR correction; Extended Data Table 1-1) during inflammatory repopulation. While acknowledging the limitations that transcriptional assessments of MMPs do not equate to protein expression or activation, there logically would be a need for proliferating and repopulating macrophages/microglia to digest growth inhibitory ECM/CAM during their migration within the injured environment. If repopulating inflammation results in a digestion of inhibitory ECM/CAM, such a condition could inadvertently augment the potential for regeneration by removal of inhibitory scar components.

While we originally hypothesized that nonresolving inflammation acts as a barrier to regeneration, our strongest evidence supports that allowing for repopulation, rather than depletion alone, may exert a more growth-permissive response to surrounding tissue. While we did reproduce the effect of PLX on axon growth into the lesion across two experimental replicates, surprisingly, we did not observe any evidence to support the ability of PTEN-KO (RFP^+^ fibers) to augment growth within the lesion. Such findings suggest that while axon growth was enhanced by PLX treatment, growth was occurring from axon subtypes that are not readily transduced by AAVrg's (see below). Importantly, because PTEN-KO was irrelevant to intralesional growth, and because both PLX-treated groups exhibited comparable effects, we collapsed groups into PLX-treated and nontreated groups to better represent the effects of PLX as a treatment.

Prior reports describe a limited ability for AAVrg's to transduce some axon subtypes, including both 5-HT^+^ and CGRP^+^ fibers (Wang et al., 2018; Blackmore et al., 2021; Metcalfe et al., 2022; Metcalfe and Steward, 2023; Stewart et al., 2023). Importantly, both axon subtypes are known to possess some potential for growth into the lesion boundaries (Inman and Steward, 2003). Our data identify that at least 5-HT^+^ and CGRP^+^ fibers respond to PLX with growth within the lesion. While we can conclude that PLX does augment axon regeneration of specific fiber types and can further conclude that depletion/repopulation does not support regeneration of AAVrg^+^ axons into the lesion, the ability for PLX to augment PTEN-KO–affected axons to grow around the lesion and through spared tissues was not, and cannot be, determined in our experimental design.

After PTEN-KO, regenerating corticospinal tract axons follow astroglial bridges or routes of spared tissue, rather than growing into the lesion core (Liu et al., 2010; Zukor et al., 2013; Du et al., 2015). From our data, we conclude that macrophages are not causal to this failure to grow through the lesion. However, we were not able to identify if a subsequent regenerative response occurred through small rims of spared tissue or through astroglial bridges. Our analysis focused on growth within the lesion and did not include regions of spared tissue that were sparse but occasionally apparent via continuous GFAP boundaries. Furthermore, as described in our prior work, while AAVrg's are emerging to be a powerful tool to target an expanded population of descending motor neurons, some limitations to the use of AAVrg's exist (Stewart et al., 2023). Specifically, spinal injections of AAVrg's target most descending axons, including spared fiber tracts, which confounds the ability to discriminate regenerated from spared axons during anatomical analyses. Second, we observed neuron cell bodies caudal to the lesion that were positive for AAVrg transduction. It is impossible to distinguish between neurons caudal to the lesion as having spared axons ascending through the lesion and into the injection site, versus the possibility that virus diffused across the lesion and labeled some neurons. Both complications challenge regenerative assessments in nonlesion compartments.

As discussed in our prior work, it is likely that the functional benefits observed after chronic PTEN-KO are elicited via spared pathways, rather than regeneration, which is supported by the speed at which recovery occurs after treatment (Stewart et al., 2023). In both our prior work, and work presented here, we observed recovery occurring in as little as 2–3 weeks posttreatment, which is likely too fast to be attributed to regeneration. While we replicated a PTEN-KO–induced recovery, neither sustained inflammatory depletion nor repopulation significantly affected locomotor abilities relative to PTEN-KO alone. Thus, while chronic neuroinflammation may play a role in regulating axon regeneration, at least within our experimental conditions, we were not able to implicate sustained inflammation as a key regulator of motor functions. The severity of our injury model, however, leaves few spared axons whereby microglial depletion at distances far away from the lesion may exert effects on sprouting or plasticity.

## Conclusions

We can conclude that sustained inflammation within chronic SCI lesions negatively regulates axon regeneration of specific fiber types and have identified that an undetermined homeostatic mechanism retains increased macrophage and microglial densities within and around chronic SCI lesions. Our results reveal a novel role of the lesion in sustaining chronic inflammation and identify nonresolving inflammation as a regulator of axon growth and regeneration.

## Data Availability

All data from this manuscript will be publicly available through the Open Data Commons for Spinal Cord Injury (www.odc-sci.org).
